# From Pandemic to Practice: How COVID-19 Has Reshaped Haemostasis in Cardiac Surgery: A Narrative Review

**DOI:** 10.3390/jcm14228109

**Published:** 2025-11-16

**Authors:** Lydia Wilkinson, Arian Arjomandi Rad, Joshua Oliver, Antonios Kourliouros

**Affiliations:** Department of Cardiothoracic Surgery, John Radcliffe Hospital, Oxford University Hospital NHS Trust, Oxford OX3 9DU, UK; lydia.wilkinson@ouh.nhs.uk (L.W.); Arian.ArjomandiRad@ouh.nhs.uk (A.A.R.); Joshua.Oliver@ouh.nhs.uk (J.O.)

**Keywords:** cardiopulmonary bypass, COVID-19, coagulopathy, anticoagulation

## Abstract

The utilisation of cardiopulmonary bypass (CPB) during cardiac surgery is often associated with complex haemostatic perturbations, frequently manifesting as a paradoxical risk of both bleeding and thrombosis. This is postulated to be driven by systemic inflammation, endothelial activation and contact activation of the coagulation cascade due to extracorporeal circulation. However, the coronavirus disease 2019 (COVID-19) pandemic revealed a unique hypercoagulable state, termed COVID-19-associated coagulopathy (CAC), also observed in those vaccinated against COVID-19. CAC displays similar physiological manifestations to those of disseminated intravascular coagulation (DIC), characterised by elevated fibrinogen and D-dimer values. The precise pathogenesis of CAC requires further elucidation though proposed mechanisms include: an exaggerated inflammatory response to COVID-19 infection or antibody proliferation due to vaccination, direct epithelial cell damage mediated by angiotensin converting enzyme 2, and ‘hypoxithrombosis’. CAC has since provided a unique framework to understand and potentially mitigate coagulation complications encountered during CPB in the post-pandemic era, as it is no longer sufficient to view COVID-19 as a transient influence on surgical risk. Rather, it must be recognized as a persistent modifier of the haemostatic environment across the population, with direct implications upon patient selection, intraoperative management and postoperative care in cardiac surgery. This review examines the pathological drivers behind CAC alongside the insights obtained from CAC management during ECMO deployment, to investigate the potential translation of such knowledge into improved anticoagulation strategies and monitoring during cardiac surgery. The use of alternative anticoagulants including factor XI inhibitors and the modulation of heparinase activity offers promising avenues to attenuate coagulopathies more commonly observed during CPB in the post-pandemic climate, whilst anti-Xa assays and viscoelastic testing have offered applicability to modern perfusion practices. By bridging the knowledge gained during the pandemic with that of conventional CPB, this review aims to inform future strategies for haemostasis management in cardiac surgery in a novel cohort of surgical patients.

## 1. Introduction

Coronavirus disease 2019 (COVID-19) is a disease of the respiratory tract, the etiological agent of which is termed severe acute respiratory syndrome coronavirus-2 (SARS-CoV-2), with over 700 million cases confirmed worldwide to date [1]. Although initial clinical presentation predominantly manifests as mild respiratory symptoms, with hallmark features such as fever, cough, and dyspnoea frequently exhibited, approximately 20% of patients develop acute respiratory distress syndrome (ARDS), often requiring advanced therapies including extracorporeal membrane oxygenation (ECMO) [2]. However, as the pandemic persisted, it became clear that COVID-19 was not solely confined to the pulmonary system; COVID-19 is now understood to be a complex multisystem disorder with profound vascular and thrombo-inflammatory implications [3].

One of the most significant and consistently reported extrapulmonary complications is COVID-19-associated coagulopathy (CAC), a distinct prothrombotic pathology characterized by dysregulated haemostasis, endothelial dysfunction, and sustained immunothrombosis. Haematologically, CAC is typified by elevated D-dimer levels, fibrin degradation products, and prolonged prothrombin times, reflecting systemic coagulation activation. Studies conducted by Tang et al. highlighted the unique nature of CAC, distinguishing it from classical disseminated intravascular coagulation (DIC), via the exhibition of preserved platelet counts and minimal consumption coagulopathy [4]. In addition, thromboembolic complications such as deep vein thrombosis (DVT) and pulmonary embolism (PE) remain frequent and severe despite the administration of prophylactic anticoagulation [5]. Such resistance to conventional anticoagulation methods underscores the unique and refractory nature of CAC, wherein standard laboratory metrics may no longer reliably reflect coagulation status or anticoagulant efficacy.

Compounding this challenge, coagulation abnormalities associated with COVID-19 are not necessarily self-limiting and confined to the acute phase of infection. Increasing evidence suggests that hypercoagulability and endothelial activation can persist for weeks to months after symptom resolution, even in patients who experienced only mild disease [6]. This post-acute sequelae of SARS-CoV-2 infection, colloquially termed ‘long COVID’, has also been linked to persistent coagulation abnormalities including ongoing thrombin generation, platelet hyperreactivity, and elevated markers of endothelial injury [7]. Such chronicity introduces a new dimension to patient risk assessment, especially in perioperative and extracorporeal settings. Intriguingly, vaccine-induced immune thrombotic thrombocytopenia (VITT), a rare but serious complication associated with adenoviral vector-based COVID-19 vaccines, also shares immunopathological features with CAC, namely, upregulated platelet activation via anti-platelet factor 4 (PF4) antibodies [8].

The broad infection rate of COVID-19 and the associated varied physiological response to infection, including CAC persistence, have commanded a profound and multifaceted impact on global healthcare systems. This has resulted in the reshaping of not only infectious disease management but also the structure and practice of surgery wherein haemostasis control is paramount, disproportionately impacting disciplines wherein haemostatic disturbances are frequently encountered such as cardiac surgery [9]. The utilisation of cardiopulmonary bypass (CPB) during cardiac surgery induces profound physiological stress, particularly upon the coagulation system, due to blood exposure to non-endothelial surfaces, consequently resulting in systemic inflammatory response syndrome (SIRS), and ischemia-reperfusion injury [10]. In patients with pre-existing CAC or post-infectious haemostatic alterations, these disturbances are exacerbated, increasing the risk of both thrombosis and haemorrhage during CPB. In order to prevent this, patients are systemically heparinised prior to CPB to mitigate thrombogenesis, and subsequently prevent clotting of the CPB circuit, with anticoagulation status monitored via activated clotting times (ACT). However, the COVID-19 pandemic has forced a re-evaluation of these practices as emerging data demonstrates an increased incidence of heparin resistance and altered anticoagulant pharmacodynamics in post-COVID patients and post-vaccine exposure [11]. This in turn promotes further haemodynamic instability during CPB, with routine ACT targets potentially no longer reflecting adequate anticoagulant effect. Therefore, the COVID-19 pandemic has resulted in a populational change in baseline coagulation status; patients with prior SARS-CoV-2 infection or vaccine exposure are now arriving in the operating room with ‘primed’ immune systems, altered endothelial responses, and unpredictable reactions to anticoagulants, with resulting erratic haemostatic implications.

A comparative analysis of the pathophysiological mechanisms and clinical management strategies in ECMO patients with CAC and those undergoing CPB reveals several similarities. In both contexts, elevated thrombotic risk persists despite prophylactic anticoagulation, driven by overlapping mechanisms such as inflammatory-mediated hypercoagulability, endothelial injury, and altered heparin responsiveness [12]. These shared vulnerabilities suggest that the targeted anticoagulation protocols, advanced monitoring techniques, and adjunctive therapies developed to manage CAC in ECMO patients may have significant translational value for optimising haemostatic management in the modern cohort of cardiac surgery patients.

This review seeks to reframe the understanding of extracorporeal-related coagulopathy from the lens of COVID-19 in the context of modern cardiac surgery and CPB, given the baseline populational coagulation changes in the era succeeding COVID-19 exposure. We therefore examine the pathophysiological drivers potentially influencing CAC alongside the implications of anticoagulant resistance. Additionally, both emerging anticoagulants and haematological monitoring strategies in cardiac surgery are detailed, aiming to inform emerging perioperative strategies to reduce thrombotic and haemorrhagic complications in the post-pandemic surgical landscape.

## 2. Materials and Methods

The authors undertook a concentrated review of the literature investigating the pathophysiology of CAC, the subsequent impact upon extracorporeal circulation, with a focus upon ECMO therapy and CPB management considerations. A comprehensive literature search was conducted across Google Scholar, PubMed/Medline and EMBASE databases from database inception to July 2025. The following search terms were applied in varying combinations: COVID-19, anticoagulation, SARS-CoV-2, thrombosis, extracorporeal membrane oxygenation, cardiopulmonary bypass, haemorrhage, coagulopathy, and acute respiratory distress syndrome. Filters were applied to restrict results to English-language, peer-reviewed publications, whilst the reference lists of included articles were also examined to identify additional studies.

Eligibility criteria were applied to relevant articles. Studies were considered eligible if original data, systematic reviews of literature, or meta-analyses on CAC, ECMO, or CPB with relevance to haemostasis or anticoagulation were reported. Among such studies, those addressing mechanisms of coagulopathy, anticoagulant pharmacodynamics, monitoring strategies, or clinical outcomes, such as bleeding, thrombosis, or heparin resistance, were included. Exclusion criteria encompassed non-English language studies, preprints without peer-review, case reports with fewer than three patients unless describing novel or mechanistically important findings, and research focused exclusively upon pediatric cohorts or non-cardiac extracorporeal therapies.

Two reviewers independently screened titles and abstracts for relevance, whilst full texts of potentially eligible studies were subsequently retrieved and assessed against the eligibility criteria. Disputes were resolved with consultation from a third reviewer. Critical appraisal of the literature was conducted by a multidisciplinary team comprising specialists in cardiopulmonary bypass and ECMO delivery and physiology, alongside practicing cardiac surgeons with extensive clinical experience and academic expertise in evidence synthesis. Given the heterogeneity of included literature, a meta-analysis was not feasible; therefore, findings were synthesised thematically across three common domains: pathophysiology of CAC, impact of CAC upon anticoagulation and emerging anticoagulants and monitoring strategies.

## 3. CAC Pathophysiology

The pathophysiology of CAC is multifactorial and reflects a convergence of innate immune activation, endothelial injury, and aberrant coagulation cascade signaling. SARS-CoV-2 gains entry into host cells primarily via angiotensin-converting enzyme 2 (ACE2) receptors, which are expressed not only in the respiratory tract but also widely distributed across the vascular endothelium [13]. Viral invasion and replication within endothelial cells trigger endothelitis and widespread endothelial activation, resulting in the upregulation of tissue factor production, disruption of antithrombotic surfaces, and loss of normal vascular integrity [14].

This endothelial insult is amplified by an exaggerated host inflammatory response marked by elevated circulating levels of interleukin 6 (IL-6), tumour necrosis factor α (TNF-α), and other pro-inflammatory mediators, termed a ‘cytokine storm’ [15]. Such cytokines potentiate platelet activation and stimulate neutrophils to release neutrophil extracellular traps (NETs), which serve as a scaffold for fibrin deposition and thus further propagate thrombogenesis [16]. The result is a sustained prothrombotic milieu which persists beyond the acute infectious phase in some patients.

A hypercoagulable state has been consistently observed in COVID-19 patients across a spectrum of comorbidities and disease severities. In a matched cohort study of 233 patients with ARDS, those with COVID-19 demonstrated a 2.7 times higher incidence of venous thromboembolism (VTE) and a 9.3 times higher incidence of pulmonary embolism (PE) compared with non-COVID ARDS patients [12]. Furthermore, a meta-analysis of 66 studies reported an overall VTE incidence of 14.1% in COVID-19 patients, rising to 45.6% amongst those admitted to intensive care units (ICU), substantially greater than in hospitalised non-COVID ARDS cohorts [17].

The rate of thrombosis within ECMO circuits is also elevated [18]. In a multicentre observational study of 150 COVID-19 patients supported with ECMO and receiving prophylactic low-molecular-weight or unfractionated heparin (loading dose 75 IU/kg, maintenance 18 IU/kg/h), Helms et al. reported that 42.6% of patients developed clinically significant thrombotic events, 16.7% of such events were PEs, and 8% were thrombotic occlusions of the centrifugal pump head [19]. Therefore, such findings underscore the elevated thrombotic burden in CAC, even despite standard anticoagulation protocols.

The spectrum of coagulation abnormalities in CAC also appears to correlate with disease severity, leading to a proposed three-stage classification [20]. Stage 1 is classified by mild physiological symptoms yet exhibits elevated D-dimer levels, compared to stage 2 which is characterised by more pronounced systemic symptoms with evidence of intravascular coagulation activation, including increased D-dimer and fibrinogen concentrations. Finally, stage 3 reflects severe systemic inflammation with a marked elevation in both D-dimer and fibrinogen concentrations occurring alongside thrombocytopenia; patients progressing to this stage generally require ICU support.

The procoagulant state observed in COVID-19 shares features with disseminated intravascular coagulation (DIC), an acquired syndrome characterised by the activation of coagulation pathways resulting in thrombosis and haemorrhage; however, it is paramount to distinguish CAC as a separate coagulopathy. Whilst both conditions present with elevated D-dimer values, CAC exhibits disproportionally elevated fibrinogen concentrations and relatively preserved platelet counts in the early and intermediate stages. Moreover, CAC does not meet the International Society on Thrombosis and Haemostasis (ISTH) diagnostic criteria for overt DIC, detailed in Table 1 [21,22]. Understanding such distinctions is critical to guide anticoagulation strategies in the context of extracorporeal circulation as conventional strategies may no longer suffice.

## 4. Sustained Hypercoagulability Following COVID-19 Infection and Vaccination: Implications for Cardiac Surgery

Emerging evidence suggests that both SARS-CoV-2 infection and COVID-19 vaccination can induce a transient yet clinically relevant hypercoagulable state, with implications extending beyond the acute phase of exposure. Patients post COVID-19 infection have presented with persistent endothelial dysfunction, elevated D-dimer levels, and increased concentrations of pro-inflammatory cytokines such as IL-6 and TNF-α, with such observations documented weeks to months after viral clearance [6,7]. Such changes reflect a sustained thrombo-inflammatory state, characterised by sustained platelet activation and impaired fibrinolysis, presenting a predisposition to microvascular thrombosis. Importantly, this state may not be readily apparent through standard coagulation screening, yet it can significantly alter haemostatic responses under surgical stress.

COVID-19 vaccination, particularly with mRNA-based platforms, has also been shown to transiently modulate coagulation dynamics. While vaccine-associated thrombosis is rare and primarily linked to adenoviral vector platforms, repeated antigenic stimulation through booster doses or prior infection can trigger a temporary inflammatory response, increasing levels of D-dimer, thrombin generation, and platelet activation markers [23,24]. These effects are typically self-limiting but may become clinically significant when superimposed within the pro-inflammatory environment of CPB.

Within the context of cardiac surgery, this evolving haemostatic landscape has led to increased reports of intraoperative heparin resistance, ACT-anti-Xa discordance, and thrombotic complications despite adherence to standard anticoagulation protocols [11]. This phenomenon is likely multifactorial, reflecting the additive effects of post-COVID endothelial injury, vaccine-primed coagulation, and CPB-induced contact activation. Given that nearly all patients present with a degree of prior SARS-CoV-2 exposure, either through infection, vaccination, or both, this shift necessitates a recalibration of perioperative anticoagulation strategies. Ultimately, recognition of the sustained hypercoagulable state induced by COVID-19 and its immunological aftermath is essential to optimise anticoagulation protocols, minimise CPB circuit-related complications, and improve outcomes in the post-pandemic surgical population.

## 5. Mechanisms of Coagulopathy Development and Implications for Cardiac Surgery

It is of paramount importance to decipher the mechanisms potentiating CAC action associated with both COVID-19 infection and immunisation, respectively, as such knowledge is essential to inform intra-operative anticoagulation management strategies and the re-evaluation of CPB practices.

### 5.1. Inflammatory Priming and CPB as a Second Hit to a ‘Primed’ System

Consistent with observations from prior SARS outbreaks, the pathogenesis of CAC reflects an interaction between the innate immune system and haemostatic pathways, termed immunothrombosis [25]. Dysregulation of this process promotes widespread microthrombi and exacerbates COVID-19-related ARDS [26]. Initial infection triggers alveolar type II pneumocyte involvement, innate cell infiltration, and a cytokine cascade (IL-1, IL-6, IL-8, IL-12, IP-10, MCP-1), with concentrations often exceeding those in SARS and non-COVID ARDS. Furthering this, neutrophil-derived cathepsin G and cytokines promote platelet aggregation, IL-6 and TNF-α upregulate tissue factor, and TNF-α elevates plasminogen activator inhibitor 1 (PAI-1), collectively suppressing tPA and impairing fibrinolysis [15,22,27].

Immunothrombosis is especially relevant when considering ECMO and CPB initiation. In those who have experienced prior COVID-19 exposure, either via infection or immunisation, the immune system may remain primed as demonstrated by sustained elevations of inflammatory markers IL-6 and TNF-α for up to 12 months post-infection [6,28,29]. Post-vaccination studies also demonstrate transient cytokine rises that typically resolve within days to weeks; however, individuals with prior infection can exhibit higher baseline inflammatory tone following immunization [30,31]. Such chronic, low-grade inflammation mirrors the cytokine response observed during CPB and may synergistically amplify the SIRS triggered by extracorporeal circulation.

CPB itself is a potent activator of both coagulation and inflammatory cascades. The exposure of blood to non-endothelial surfaces initiates contact activation via the factor XII pathway whilst ischaemia-reperfusion injury and complement activation contribute to a systemic inflammatory surge. Mechanistic and translational investigations consistently demonstrate perioperative rises in PAI-1 alongside impaired fibrinolysis and increased tissue factor expression on monocytes and endothelium, correlating with thrombotic events and transfusion requirements [10,32,33,34,35,36]. These changes promote thrombin generation, impair fibrinolysis, and exacerbate platelet-endothelium interactions. Thrombin–antithrombin complexes, elevated fibrinogen, and viscoelastic evidence of hypercoagulability are frequently observed during and after CPB [37,38]. In post-COVID patients, this cascade is superimposed upon a vascular system primed toward thrombosis, the consequence of which is amplified platelet activation, NET formation, and endothelial reactivity. Elevated circulating NETs and platelet–neutrophil aggregates, together with endothelial dysfunction, have been associated with increased thrombotic risk despite prophylaxis in severe COVID-19 [12,16,39]. This results in a hyperreactive coagulation system wherein immunothrombosis may persist and resurface peri- and postoperatively, thus presenting as an unpredictable coagulation profile.

CPB also alters immunity and coagulation parameters in vaccinated individuals. Hayashi et al. reported a marked reduction in SARS-CoV-2 spike protein antibody titres following CPB, with antibody preserved rates (APR) dropping from 0.80 in non-CPB patients to 0.46 in those undergoing bypass, suggesting that CPB may transiently suppress adaptive immune responses [40]. Strobel et al. similarly found that COVID-19 vaccine antibody concentrations declined significantly on postoperative day one following CPB, before recovering at one month, highlighting the immunomodulatory effects of extracorporeal circulation [41]. While these studies focus on humoral immunity, they underscore the broader immunological disruption induced by CPB, which may interact with pre-existing inflammatory priming to exacerbate haemostatic dysregulation.

### 5.2. Angiotensin Converting Enzyme 2 (ACE2)-Mediated Epithelial and Endothelial Injury

ACE2 is the specific functional receptor for SARS-CoV-2 and is prevalent within the respiratory system, expressed abundantly on alveolar epithelial cells and to a lesser extent on oral and nasal mucosa, demonstrating that the lungs are the primary target of SARS-CoV-2 [13]. ACE2 hydrolyses pro-inflammatory angiotensin-2 into angiotensin (1–7), which exerts a vasodilatory and anti-inflammatory effect via Mas-related G-protein-coupled receptors [42]. However, ACE2 expression is downregulated by SARS-CoV-2 viral binding, reducing angiotensin (1–7) and increasing angiotensin-2 expression as a direct result [43]. Elevated angiotensin-2 concentrations stimulate immune and parenchymal cells to increase TNF-α, IL-6 and tissue factor production, activating the extrinsic coagulation pathway. Tissue factor-activated factor VII (VIIa) also triggers the upregulation of protease-activated receptor signalling, which induces further endothelial injury [44].

Endotheliopathy initiates both microthrombotic and inflammatory cascades. Endothelial activation promotes platelet activation and exocytosis of uncharacteristically large von Willebrand factor multimers (ULVWF), which form ULVWF-platelet complexes that tether to injured endothelium, driving microvascular thrombosis [45]. Such processes underpin the development of CAC and contribute to pulmonary microthrombi, PE and DIC [24]. Importantly, SARS-CoV-2-mediated endotheliopathy and epithelial injury may persist beyond viral clearance, with widespread microvascular damage altering the haemostatic balance between anticoagulant and procoagulant states. This results in a predisposition to both thrombosis and bleeding, particularly in patients undergoing extracorporeal therapies.

In the context of cardiac surgery, epithelial cell damage has significant implications, especially when occurring within the pulmonary microvasculature. Alveolar epithelial injury compromises the integrity of the alveolar-capillary barrier, increasing vascular permeability and promoting interstitial oedema. This not only impairs gas exchange but also facilitates translocation of inflammatory mediators and activated leukocytes into the systemic circulation, amplifying SIRS triggered by CPB. However, CPB itself can also further exacerbate endothelial shedding and cytokine release by inducing mechanical trauma experienced due to arterial roller pump exposure [10]. Therefore, the effect of pre-existing epithelial and endothelial injury combined with the aforementioned CPB-induced perturbations creates a uniquely vulnerable haemostatic environment. The pulmonary epithelium, already compromised, may fail to buffer the inflammatory surge and coagulation activation associated with CPB, contributing to increased rates of postoperative pulmonary dysfunction, refractory bleeding, and thrombotic complications.

### 5.3. Hypoxithrombosis and Enzymatic Heparin Degradation

Emerging evidence implicates hypoxia as an important contributor to thromboembolic risk in COVID-19, independent of systemic inflammation. The Gemelli Against COVID-19 project reported increased instances of VTE among non-ICU patients with respiratory insufficiency, despite pharmacological thromboprophylaxis, a pattern termed ‘hypoxithrombosis’ [46]. However, within this cohort, DVT cases also differed from non-DVT cases by raised D-dimer (>3000 µg/L), current or prior malignancy, and requirement for high-flow nasal oxygen >8 L/min, indicating that hypoxia was not the only associated factor. Nonetheless, hypoxia remains mechanistically and epidemiologically linked to thrombosis; silent or subclinical hypoxaemia has been documented in ambulatory COVID-19 patients which can prime endothelial, platelet, and coagulation pathways toward a prothrombotic state. Hence, the Gemelli findings do not exclude hypoxia’s role, rather suggesting that hypoxia often acts alongside other risk factors to increase VTE risk, and that unrecognised hypoxaemia may result in patients presenting for cardiac surgery with a haemostatic system predisposed to thrombosis.

Although the mechanistic background of hypoxithrombosis remains incompletely defined, parallels may be drawn from altitude physiology wherein chronic hypoxia and secondary polycythaemia have demonstrated an increased VTE risk [47]. Sustained hypoxia activates hypoxia-inducible transcription factors (HIFs), which regulate cellular adaptation to low oxygen tension by upregulating PAI-1, and suppressing fibrinolysis, thereby promoting a prothrombotic state [48]. Clinical data reinforces this association; the Tromsø Study identified that COPD patients with SaO_2_ < 96% had a 1.5 times increased risk of VTE compared to normoxic controls, while Klok et al. reported a 77% increase in thrombotic events in mechanically ventilated COVID-19 patients with SaO_2_ < 90% [49,50]. Although immobility contributes via Virchow’s triad, hypoxia itself appears to exert a direct procoagulant effect. Interventions such as prone positioning, which improves oxygenation (SaO_2_ 94% vs. 80%), may mitigate this risk [51].

Hypoxia may also impair anticoagulant efficacy. The activity of both heparinase, an enzyme which degrades both low-molecular-weight and unfractionated heparin, and heparanase, similarly cleaving heparan sulphate and heparin chains, are upregulated under hypoxic conditions [52]. Wu et al. demonstrated increased heparanase expression and activity in MIA PaCa-2 pancreatic cancer cells exposed to 1% oxygen [53]. Glycocalyx degradation is also mediated by heparanase which removes the antithrombotic surface of the endothelium, exposing subendothelial matrices and releasing procoagulant microparticles. Taken together, these findings suggest that hypoxia-driven upregulation of heparinase and heparanase not only undermines the anticoagulant effect of administered heparin but also destabilises endogenous vascular anticoagulant mechanisms.

Persistent hypoxaemia in post-COVID patients may induce upregulated heparinase and heparanase activity, manifesting as diminished ACT values and necessitating increased heparin requirements for extracorporeal support [11]. CPB itself induces haemodilution, hypothermia, and non-physiological flow which exacerbates tissue-level hypoxia and further stimulates enzymatic activity. This creates a positive feedback loop in which hypoxia drives both thrombogenesis and undermines anticoagulant efficacy, creating an unstable haemostatic environment.

### 5.4. Vaccine-Associated Hypercoagulability

Vaccination against SARS-CoV-2 produces reproducible, short-lived activation of the innate immune system, and measurable shifts in haemostatic biomarkers including transient rises in fibrinogen, factor VIII, vWF and indicators of platelet activation [54]. Mechanistic studies have also implicated type I interferon to Toll-like receptor (TLR) signalling, and monocyte–endothelial crosstalk as further plausible drivers of a procoagulant state [55]. Large surveillance and registry analyses, however, have not demonstrated a population-level increase in clinical thrombotic events attributable to vaccination, whereas mechanistic and single-centre biomarker studies which present transient procoagulant signals are generally small, heterogeneous in design, often underpowered for clinical endpoints, and subject to confounding by prior infection and comorbidity. Cardiac surgery with cardiopulmonary bypass imposes profound haemostatic stress which independently elevates thrombin generation and suppresses fibrinolysis. Therefore, it is biologically plausible that a temporally overlapping, vaccine-related procoagulant shift could act additively to reduce the threshold for perioperative thrombosis or consumptive coagulopathy in susceptible individuals such as those with recent infection, persistent biomarker abnormalities, active malignancy, advanced age, or endothelial dysfunction. In the absence of robust prospective perioperative data, the appropriate clinical stance is one of calibrated vigilance rather than prescriptive change.

Mechanistically, vaccination elicits rapid innate immune activation through pattern recognition receptors, TLR7/8 for mRNA vaccines and TLR9 or adenoviral capsid sensing for vector platforms, driving type I interferon and NF-κB signalling with downstream release of IL-6 and other acute phase cytokines [56]. This cytokine storm transiently increases hepatic synthesis of fibrinogen and factor VIII, elevates vWF via Weibel-Palade body exocytosis, and upregulates PAI-1. Collectively such shifts enhance thrombin generation whilst suppressing fibrinolysis [57]. Endothelial priming adds further prothrombotic cues, including increased tissue factor expression on activated monocytes and endothelial cells and the shedding of tissue factor-bearing microparticles, which amplify extrinsic pathway activation under surgical stress.

Platelet reactivity is also briefly augmented post-vaccination, with increased P-selectin expression, release of platelet factor 4 (PF4), polyphosphates, and procoagulant extracellular vesicles. PF4 binds to heparin and endogenous glycosaminoglycans, locally neutralising heparin’s antithrombin effect and facilitating dense fibrin network formation. Polyphosphates and extracellular histones further potentiate factor XII-dependent contact pathway activation, a mechanism particularly relevant during CPB wherein blood-surface interactions already trigger kallikrein-kinin cascades [8]. NETs induced by post-vaccination cytokines provide a scaffold for platelets and fibrin, concentrate activated coagulation enzymes, and inhibit fibrinolysis via histone-mediated plasminogen inactivation. In rare cases, adenoviral vector vaccines have been associated with VITT, mediated by anti-PF4 IgG immune complexes activating platelets through FcγRIIa [58]. More commonly, however, non-pathogenic anti-PF4 antibodies or simply elevated PF4 levels can subtly shift perioperative haemostasis without overt thrombocytopenia.

These transient vaccine-induced changes are clinically relevant when superimposed upon the pro-inflammatory and pro-coagulant environment of CPB. Contact pathway activation and complement-leukocyte crosstalk synergise with vaccine-primed platelets and NETs, increasing the susceptibility for circuit thrombosis and elevated heparin dose requirements, particularly when antithrombin is diluted or consumed during CPB. Such potential drivers of CAC are summarised in Table 2.

## 6. CAC Management Considerations Within Cardiac Surgery

The COVID-19 pandemic did not just affect a subset of critically ill patients, instead leaving an imprint on the haemostatic and endothelial health of the global population. As a result, the field of cardiac surgery, particularly procedures requiring CPB, must adapt to a more complex and unpredictable haemostatic landscape. A generic approach to anticoagulation is no longer sufficient within a population demonstrating variable thrombotic and bleeding risk profiles. Therefore, future perioperative practice must consider alternative agents and adjunctive strategies to control inflammation and thrombosis, employ multimodal anticoagulation monitoring, and stratify risk based on prior infection and comorbidities.

### 6.1. Emphasis on Elevated Oxygen Delivery

Hypoxithrombosis presents several complications to cardiac surgery by priming the coagulation cascade in a prothrombotic state, subsequently exacerbated by the initiation of CPB, promoting a positive feedback loop of thrombogenesis. In the perioperative setting, this translates into increased heparin requirements, ACT-anti-Xa discordance, and a greater risk of both thrombotic and bleeding complications. Early initiation of extracorporeal support strategies, such as ECMO, has been proposed to mitigate hypoxia-driven coagulopathy. The 2018 EOLIA trial examined the impact of early ECMO initiation in ARDS patients (PaO_2_/FiO_2_ 150 mmHg) compared to delayed initiation at PaO_2_/FiO_2_ < 80 mmHg [61]. Although the primary endpoint of 60-day mortality reduction was not statistically significant (35% vs. 46%), high crossover (28%) from the control to ECMO arm likely diluted the effect. Post-hoc Bayesian analyses and subsequent meta-analyses supported the interpretation that early ECMO initiation may benefit selected patients [61,62]. Importantly, bleeding and thrombotic complications were lower in those initiated on ECMO pre-hypoxemia compared to those cannulated later, despite similar anti-Xa activity [61]. Translating this to cardiac surgery, active intraoperative strategies to minimise hypoxaemia before and during CPB must be paramount and will be discussed further.

Mechanistically, tissue-level hypoxia activates HIF-1α and HIF-2α, upregulates tissue factor and PAI-1, and promotes NETosis, creating a procoagulant environment that can be amplified by CPB wherein haemodilution, hypothermia and non-physiological flow further impair microcirculatory oxygen delivery. Therefore, maintaining adequate indexed oxygen delivery (DO_2_i) during CPB is a physiologically coherent strategy to limit hypoxia-driven coagulation activation. Contemporary perioperative and perfusion guidance therefore emphasises goal-directed perfusion to maintain adequate indexed DO_2_ by minimizing periods of reduced arterial pump flow, active temperature control and minimisation of unnecessary prime volume rather than fixed haemoglobin targets [63,64]. The haemodilution paradox is central to CPB management as reducing haematocrit reduces blood viscosity, which may improve capillary perfusion and reduce sludging in the context of mild hypothermia. However, it simultaneously reduces arterial oxygen content and therefore DO_2_; thus, microcirculatory perfusion may increase though oxygen delivery can decline unless pump flow or haemoglobin are adjusted to compensate. Such approaches improve lactate and SvO_2_ as surrogates of tissue oxygenation and are now advocated by guideline groups [65,66]. However, direct evidence linking elevated intraoperative DO_2_ to reduced thrombotic events in post-COVID or post-vaccine cardiac surgical cohorts is lacking and is derived mainly from mechanistic inference and organ-protection studies rather than thrombosis-specific endpoints [67]. Studies are observational, heterogeneous in DO_2_ thresholds and timing, underpowered for thrombotic endpoints, and susceptible to confounding by prior infection, comorbidity and perfusion practice variability. Implementation challenges including protocol adherence, monitoring technology and human factors are described and may influence effectiveness in routine practice. These strategies may theoretically attenuate tissue-level hypoxia and the associated coagulopathic effects, improving outcomes in post-COVID and high-risk surgical patients. However, further research directly linking elevated intraoperative DO_2_ to fewer thrombotic endpoints in such cardiac surgical cohorts is required.

### 6.2. Alternative Anticoagulants

Achieving systemic anticoagulation via the administration of unfractionated heparin remains the gold standard for anticoagulation during CPB, due to its rapid onset, reversibility with protamine sulfate, and cost-effectiveness. However, the COVID-19 experience has revealed limitations in heparin efficacy, manifesting as heparin resistance reported in up to 9.2% of adult cardiac surgery patients undergoing CPB during the pandemic [68]. Mechanistically, this resistance is multifactorial, primarily driven by reduced antithrombin III levels, owing to consumption during systemic inflammation and CPB. However, in the context of CAC, this may also occur in the presence of heparin-binding proteins overexpressed in COVID-19, such as platelet factor 4, fibrinogen, and C-reactive protein, alongside the enzymatic degradation of heparin by heparanase and heparinase [69]. These observations underscore the vulnerability of unfractionated heparin in inflammatory states such as CAC, prompting the exploration of alternative anticoagulants and adjunctive strategies that could be used selectively in cardiac surgery.

Direct thrombin inhibitors (DTIs), including bivalirudin and argatroban, are attractive alternatives to heparin in CAC as they act independently of antithrombin and directly inhibit both circulating and clot-bound thrombin. This pharmacological profile is particularly relevant in patients with heparin resistance, antithrombin deficiency, or heparin-induced thrombocytopenia (HIT), all of which have been reported with increased frequency in the post COVID-19 era [68]. Bivalirudin use has been associated with reduced major bleeding, lower transfusion requirements, and fewer thrombotic complications when utilising bivalirudin compared to heparin, whilst argatroban has shown efficacy in COVID-19 patients with HIT reporting fewer bleeding complications than heparin [70,71,72]. Despite these advantages, DTIs have important limitations in the context of CPB. Unlike heparin, DTIs do not inhibit the contact activation pathway, a major driver of thrombin generation, which is strongly upregulated during CPB and potentially further amplified in CAC. Moreover, DTIs lack a specific reversal agent, complicating haemostasis at the time of separation from CPB, a particularly acute concern in the prothrombotic yet bleeding-prone milieu of CAC. Moreover, evidence derived from non-surgical environments, such as the RE-LY trial of the DTI dabigatran, demonstrated increased rates of gastrointestinal bleeding compared to warfarin, with subsequent analyses reporting elevated perioperative bleeding risk [73,74]. While these findings cannot be directly extrapolated to parenteral DTIs, they underscore the need for caution when considering DTIs as primary anticoagulants in high-risk surgical settings. Therefore, whilst DTIs may be selectively valuable in CAC patients presenting with HIT, heparin resistance or profound antithrombin deficiency, their broader role across CPB remains ill-defined.

A more mechanistically aligned approach to CAC is the inhibition of factor XI (FXI), a key driver of thrombin generation via activation of the contact pathway. FXI is relevant in the CAC context wherein hyperinflammation and endothelial injury amplify contact activation. Patients with congenital FXI deficiency exhibit reduced rates of thrombotic risk without incurring major bleeding, and early-phase studies of FXIa inhibitors such as [75] frunexian (EP-7041) demonstrate promising pharmacokinetics and reduced VTE incidence with minimal bleeding [75,76,77,78]. When applying such findings to CPB, FXI inhibition could theoretically suppress circuit-induced contact activation whilst also preserving haemostasis, which is a paradigm shift compared with heparin, though translation to CPB remains theoretical as no surgical trial data currently exists.

COVID-19 has revealed the role of heparanase and heparinase in degrading both endogenous heparan sulfate and exogenous heparin, contributing to heparin resistance. Defibrotide has been shown to inhibit the effect of heparanase-1 upon heparin degradation at concentrations of >125 μg/mL, thereby potentiating its anticoagulant effect [52,79,80]. A further therapeutic avenue is the use of roneparstat, a competitive heparinase inhibitor with additional antiviral and anti-inflammatory properties. Xiang et al. demonstrated that roneparstat reduced SARS-CoV-2 infectivity and attenuated cytokine release from macrophages in vitro [81]. By preserving endothelial glycocalyx integrity, roneparstat could theoretically mitigate heparin resistance and reduce thrombo-inflammation during CPB. However, though such agents demonstrate a potential effect upon CAC during CPB, they remain experimental and require further elucidation in human clinical trials.

### 6.3. Anticoagulation Monitoring

The demographic of cardiac surgical patients has become more heterogeneous from a haematological perspective, and reliable assessment of coagulation status has become increasingly important. The postoperative period remains one of the highest risk windows for both bleeding and thrombosis, yet conventional coagulation testing often fails to capture the dynamic, multifactorial nature of haemostatic disturbances induced during surgery in the context of CAC.

ACT testing remains the ‘gold standard’ for measuring heparin-mediated anticoagulation, due to the ease of test implementation, accuracy and rapid results. However, ACT is influenced by hypothermia, haemodilution, thrombocytopenia, and inflammatory proteins, all of which are common during CPB and exaggerated in COVID-19 patients [82]. Importantly, ACT prolongation does not preclude thrombus formation, with multiple reports of CPB circuit thrombosis occurring despite ACT values >400 s [83]. Recent case reports have highlighted that ACT may be falsely elevated in patients with recent COVID-19 infection, leading to underdosing of heparin and risking catastrophic circuit clotting [84]. Similarly, activated partial thromboplastin time (aPTT) and PT/INR provide only isolated snapshots of the intrinsic and extrinsic pathways, respectively, and are poorly predictive of bleeding or thrombosis in the perioperative period. These limitations underscore the need for multimodal monitoring strategies that can account for the complex interplay of inflammation, platelet activation, and fibrinolysis characteristic of CAC. The use of viscoelastic testing, for example, conducted during point of care tests, may also be undertaken to form a broader clinical picture of anticoagulation and coagulation pathway inhibition to prevent prothrombotic states. Moreover, the use of alternative, non-heparin, anticoagulants generally requires additional monitoring, which takes longer to produce results, such as the employment of anti-Xa monitoring to assess direct anti-Xa inhibitors including apixaban. In such instances, a picture of anticoagulation values should be built up initially utilising a variety of tests, and deviations from such a baseline can be utilised to indicate coagulopathy development rather than test ranges. Emphasis on the development of rapid point of care testing for alternative anticoagulants is paramount.

Anti-Xa assays directly quantify the inhibition of factor Xa by heparin or LMWH and are more precise than ACT, particularly in cases of heparin resistance or antithrombin deficiency. Khaja et al. demonstrated that aPTT correlated more closely with anti-Xa concentrations than ACT in ECMO patients with suspected heparin resistance, highlighting the limitations of ACT as a sole monitoring modality [85]. A systematic review by Willems et al. including over 2000 ECMO patients identified that anti-Xa-guided anticoagulation was associated with fewer bleeding events and lower mortality compared with time-based strategies [86]. Therefore, in the current surgical population where elevated fibrinogen and heparinase activity may confound ACT values, anti-Xa monitoring provides a more accurate reflection of anticoagulant effect. However, its longer turnaround time at 15–30 min limits intraoperative utility, underscoring the need for point-of-care anti-Xa platforms.

Viscoelastic assays such as thromboelastography (TEG) and rotational thromboelastometry (ROTEM) provide real-time assessment of clot formation, strength, and fibrinolysis, offering a comprehensive picture of haemostasis [87]. Chandel et al. reported that D-dimer values, traditionally used as markers of thrombosis, had an inverse relationship with hypercoagulability as measured by TEG maximum amplitude (MA), while fibrinogen correlated positively [88]. A fibrinogen concentration >441 mg/dL predicted a TEG MA ≥ 68 mm with 91% sensitivity, suggesting that fibrinogen may be a more reliable marker of hypercoagulability than D-dimer in the post-COVID-19 context by reflecting the severity of CAC in cardiac surgery patients rather than identifying patients likely to benefit from additional anticoagulation. Fibrinogen concentration can therefore be utilised to monitor adequate anticoagulation alongside ACTs which also reflects the activity of heparinase; increased activity will degrade heparin faster and thus be mirrored in diminished ACTs, giving an early indicator of potential thrombus formation. Stradleigh et al. compared TEG with standard heparin monitoring assays in COVID-19 positive ECMO patients and found poor correlation between ACT, aPTT, anti-Xa, and TEG parameters [84]. Importantly, TEG with platelet mapping (TEG-PM) provided a more accurate representation of overall haemostasis, including platelet dysfunction and fibrinolysis shutdown, which were not captured by conventional assays. These findings support the integration of viscoelastic testing into perioperative monitoring protocols for post-COVID cardiac surgery patients. ROTEM has also been shown to rapidly quantify fibrinogen contribution to clot firmness and detect COVID-19-induced hypercoagulability within 10 min [89]. In the intraoperative setting, ROTEM can guide heparin and protamine dosing, identify fibrinolysis shutdown, and detect microthrombi formation, all of which are critical in the management of post-COVID patients undergoing cardiac surgery.

Platelet function assays such as Multiplate^®^ and VerifyNow^®^ provide rapid assessment of platelet reactivity to agonists including ADP and collagen. COVID-19 patients frequently demonstrate platelet activation and consumption, with residual hyperreactivity contributing to thrombotic risk [89]. In cardiac surgery, platelet function testing is particularly valuable in patients with recent COVID-19 exposure or vaccination, those on dual antiplatelet therapy, or those with renal dysfunction, within which platelet dysfunction may exacerbate bleeding or thrombosis despite normal platelet counts.

Novel biomarkers are under investigation to capture the endothelial and immunothrombotic dimensions of CAC. Syndecan-1 and soluble thrombomodulin are core indicators of endothelial glycocalyx degradation and endothelial injury. In severe COVID-19, elevated syndecan-1 levels correlate with decreased survival indices and track with thrombomodulin and inflammatory cytokines, implicating pervasive endothelial disruption in disease progression [90]. Prospective data further show early increases in syndecan-1 and thrombomodulin with parallel rises in angiogenic factors, including angiopoietin-2, VEGF and HGF, linking endothelial damage to dysregulated repair responses [91]. Cardiac surgery and CPB similarly shed the glycocalyx; perioperative rises in syndecan-1 have been associated with microvascular leak, inflammation, and coagulopathy, suggesting that preexisting COVID-related endotheliopathy may compound CPB-induced injury and heighten thrombotic risk [92]. Practically, perioperative syndecan-1 and thrombomodulin screening may therefore identify patients presenting with fragile endothelial layers, prompting tighter monitoring of DO_2_ targets, earlier antifibrinolytic stewardship, and improved vigilance for microthrombi.

## 7. Conclusions and Future Directions

The COVID-19 pandemic has exposed significant vulnerabilities in current anticoagulation strategies for CPB, with CAC amplifying heparin resistance through antithrombin depletion, heparin-binding proteins, and heparinase activity. While heparin remains indispensable, alternative agents have been explored. DTIs such as bivalirudin and argatroban provide antithrombin-independent anticoagulation and have demonstrated selective benefits in heparin resistance and HIT, though their lack of reversal agents, failure to inhibit contact activation, and bleeding risks limit broader use. These shortcomings highlight the need for comparative studies in CAC populations to clarify whether DTIs can be safely and effectively deployed in defined clinical scenarios. Similarly, factor XI inhibition represents a mechanistically attractive approach, particularly given the central role of contact activation in CAC, with early data on frunexian (EP-7041) suggesting reduced thrombosis without major bleeding. Yet, the absence of surgical validation underscores the importance of targeted clinical trials in CPB to determine whether this theoretical advantage translates into practice. Adjunctive therapies such as defibrotide and roneparstat, which inhibit heparinase-mediated heparin degradation, directly address CAC-specific mechanisms, but their potential will only be identified through translational studies which establish dosing, safety, and efficacy in surgical settings.

Equally, the pandemic has emphasised that pharmacological innovation must be matched by advances in monitoring. Conventional assays such as ACT and aPTT are often unreliable in CAC, wherein inflammatory mediators distort coagulation profiles. Emerging modalities, including viscoelastic testing, anti-Xa monitoring, and biomarker-integrated point-of-care assays, offer the potential for more accurate, real-time assessment of anticoagulant effect, though require systematic evaluation in surgical populations to define thresholds and clinical utility. Thus, the future of anticoagulation in cardiac surgery after COVID-19 lies not only in the development of novel agents but also in the refinement of monitoring strategies that can guide their safe and effective use. Together, these avenues point towards a multimodal, personalised approach that moves beyond reliance on heparin alone and is tailored to the unique prothrombotic and inflammatory environment of CAC.

## Figures and Tables

**Table 1:** Normal haemotological parameters of platelet count, fibrinogen, prothrombin time prolongation and D-dimer tests with the associated deviations across disseminated intravascular coagulation and COVID-19-associated coagulopathy.

Haematological Parameter	Normal Range in Adults	Disseminated Intravascular Coagulation	COVID-19-Associated Coagulopathy	References
Platelet count (×10^9^/L)	150–400	↓	Normal range or mildly ↓	[21,22]
Fibrinogen (g/L)	2.0–4.0	↓	↑ (often >5.0)	[20,21]
PT prolongation (s)	<3 above control	↑	Normal range or mildly ↑	[12,21]
D-dimer (ng/L FEU)	<500	↑↑ (often >1000)	↑↑↑ (often >3000; severe cases >5000)	[19,22]

**Table 2:** Mechanisms of COVID-19-associated coagulopathy development and the subsequent clinical impact upon coagulation. Proposed methods to prevent the development of such coagulopathies are also detailed.

Pathophysiological Driver	Mechanism	Manifestation in Coagulopathy	References
Inflammatory priming and CPB as a second hit to a ‘primed’ system	Cytokine storm induces neutrophil extracellular trap (NET) formation, platelet hyperactivity, and monocyte–platelet aggregation; IL-6 and TNF-α upregulate tissue factor and suppress fibrinolysis.	Arterial and venous thrombosis; platelet and coagulation factor consumption may lead to secondary haemorrhage.	[15,22,25,26,27,28,29]
Angiotensin converting enzyme 2 (ACE2)-mediated epithelial and endothelial injury	SARS-CoV-2 binds to ACE2 receptors on pulmonary epithelial and endothelial cells, triggering endotheliopathy and activation of microthrombotic and inflammatory pathways.	Platelet activation and exocytosis of ultralarge von Willebrand factor (ULVWF) multimers; ULVWF–platelet complexes anchor to injured endothelium, promoting microvascular thrombosis and DIC.	[13,42,43,44,45]
Hypoxithrombosis and enzymatic heparin degradation	Hypoxia upregulates hypoxia-inducible transcription factors (HIFs), promoting expression of prothrombotic mediators (e.g., PAI-1, tissue factor); also induces heparinase and heparanase activity.	Elevated D-dimer and fibrinogen; polycythaemia; widespread microthrombi; reduced efficacy of heparin due to enzymatic degradation, contributing to anticoagulation resistance.	[11,46,47,48,49,50,51,52,53]
Vaccine-associated hypercoagulability	mRNA and adenoviral vector vaccines activate innate immune pathways (TLR7/8, TLR9, type I interferon, NF-κB), elevating fibrinogen, factor VIII, vWF, and PAI-1. Platelet activation is enhanced via P-selectin, PF4 release, polyphosphates, and extracellular vesicles. NETs provide a scaffold for coagulation enzymes and fibrin. Rarely, adenoviral vaccines trigger VITT through anti-PF4 IgG immune complexes activating platelets via FcγRIIa.	Transient hypercoagulability with increased thrombin generation, impaired fibrinolysis, and perioperative heparin resistance. VITT may manifest as thrombocytopenia with cerebral or splanchnic thrombosis.	[8,54,55,56,57,58,59,60]

## Abbreviations

The following abbreviations are used in this manuscript:

ACE2	Angiotensin-converting enzyme 2
ACT	Activated clotting time
aPTT	Activated partial thromboplastin time
ARDS	Acute respiratory distress syndrome
CAC	COVID-19-associated coagulopathy
CPB	Cardiopulmonary bypass
DIC	Disseminated intravascular coagulation
ECMO	Extracorporeal membrane oxygenation
ICU	Intensive care unit
ISTH	International Society on Thrombosis and Haemostasis
NET	Neutrophil extracellular traps
PAI-1	Plasminogen activator inhibitor 1
PE	Pulmonary embolism
PF4	Platelet factor 4
ROTEM	Rotational thromboelastometry
TEG	Thromboelastogram
TNF	Tumur necrosis factor
SARS-CoV-2	Severe acute respiratory syndrome coronavirus-2
VTE	Venous thromboembolism
VITT	Vaccine-induced immune thrombotic thrombocytopenia
